# Can Working Memory Task-Related EEG Biomarkers Measure Fluid Intelligence and Predict Academic Achievement in Healthy Children?

**DOI:** 10.3389/fnbeh.2020.00002

**Published:** 2020-01-22

**Authors:** Wei Luo, Renlai Zhou

**Affiliations:** Department of Psychology, Nanjing University, Nanjing, China

**Keywords:** event-related potentials, event-related synchronization, fluid intelligence, academic achievement, machine learning

## Abstract

**Background:**

Educational psychology research has linked fluid intelligence (Gf) with working memory (WM), but it is still dubious whether electroencephalography (EEG) markers robustly indicate Gf. This study addresses this issue and notes the relationship between WM task-related EEG markers with Gf and academic performance.

**Method:**

A sample of 62 healthy children between the ages of 9 and 12 years was selected to perform three tasks: (1) Raven’s Standard Progressive Matrices (RSPM) test to assess Gf; (2) 2-back task to assess central executive system (CES); and (3) delayed match-to-sample task to assess short-term storage. These subjects were divided into high ability (HA) and low ability (LA) groups based on their RSPM scores. Support vector machine and logistic regression were used to train the EEG candidate indicators. A multiple regression was used to predict children’s academic performance using P3 amplitude, P2 latency, and θ-ERS.

**Results:**

Behavioral results demonstrated that the correct rate of the HA group is higher than that of the LA group. The event-related potential results of the 2-back task showed that the P3 amplitude of the HA group was relatively larger and that the P2 latency was shorter than that observed in the LA group. For the delayed matching to sample task, the θ-ERS of the LA group was higher than that of the HA group. However, the area under the curve of these three indicators for Gf was < 0.75 for each and < 0.85 for the combined indicators. In predicting academic performance, only P3 amplitude showed a significant effect.

**Conclusion:**

These results challenge previous findings, which reported that P3, P2, or theta power might be used in standard psychometric tests to assess an individual’s intelligence.

## Highlights

- Both executive and storage components of working memory were considered in this research at the EEG level.
- Both logistic regression and support vector machine classifiers were applied to train the EEG data from working memory tasks to classify the fluid intelligence (Gf) of children.
- Single (area under curve [AUC] < 0.75) or combined (AUC < 0.85) indicators of three EEG signals (P3 amplitude/P2 latency/θ-event-related synchronization) all showed moderate AUC in the receiver operating characteristic analysis of Gf.
- A multiple linear regression of EEG markers to children’s academic achievement showed that only the P3 amplitude exhibited an effect (β = 0.017, *p* = 0.035).

## Introduction

With the development of artificial intelligence (AI), exploring the relationship between neurophysiological markers and psychological characteristics has become intensified. Especially in the fields of information engineering, facial expression recognition, and smart surgery, an integrated automatic identification system based on biomarkers is gradually being established. By collecting and analyzing the information of specific groups of index, it is possible to recognize and diagnose certain characteristics, abilities, or attributes of an organism. Relevant studies from the interdisciplinary fields of medicine and cognitive neurology have indicated that multiple brain activity markers extracted from EEG results can be good indicators of the state of consciousness or the cognitive state of human beings (Missonnier et al., 2007; Sitt et al., 2014; Engemann et al., 2018). Specially, compared with other biomarkers, EEG biomarkers have the advantages of economy, convenience, and efficiency. Combined with machine learning, EEG biomarkers can automatically identify and classify various clinical patients, so they represent the preferred clinical indicators for predicting treatment response (Engemann et al., 2018).

Fluid intelligence (Gf) has always been the focal topic in cognitive psychology, as well as in recent years. In many cases, such as career counseling or clinical application, it is necessary to assess a person’s level of intelligence. However, presently, the intelligence test is still based on a pencil-and-paper test; the era of intellectualization has introduced new requirements for assessing intelligence. Intelligence scales such as Raven’s and Wechsler’s have demonstrated good reliability and validity; even when the testing method is relatively simple, these intelligence scales are widely used in general intelligence tests; however, when it comes to the plasticity of Gf and the evaluation of robot intelligence, these methods appear to be insufficient. How do we develop a scientific evaluation system based on neurocognition? How do we carry out targeted intelligent shaping based on the working mechanism of the brain? Obviously, to solve these problems, the neural basis of Gf warrants further clarification. In addition, with the demand of AI for intelligence shaping and people’s expectations for improving Gf, the current ways of intelligence assessment are facing new challenges: “Knowing wisdom and making intelligence, knowing intelligence and making evaluation” requires cognitive neuroscience to make further breakthroughs in the understanding of Gf and develop a more reliable evaluation system. Several previous studies have applied machine learning methods to explore EEG signals that were effective in verifying Gf (Neubauer and Fink, 2009; Itthipuripat et al., 2013; Wronka et al., 2013; Amin et al., 2015; Dong et al., 2015; Qazi et al., 2017; Wongupparaj et al., 2018), and some of them revealed that individuals with different Gf levels can be well distinguished (Amin et al., 2015; Qazi et al., 2017).

Amin et al. (2015) conducted research on 34 healthy adults (ranging in age from 20 to 30 years) using the visual oddball task. An analysis of P3 component induced by the visual oddball task showed that P3 amplitude could significantly predict individual scores on Raven’s Advanced Progressive Matrices with an area under the curve (AUC) reaching 0.82. Therefore, P3 amplitude could be used as a good supportive index in the standard psychological test for evaluating an individual’s learning or memory ability (Amin et al., 2015). This study is the first to test the EEG effect in measuring Gf. Subsequently, Qazi et al. (2017) also used the visual oddball paradigm as a tool to examine Gf (marked by Raven’s Advanced Progressive Matrices scores) and used the support vector machine (SVM) classifier to test the discriminant ability of delta band to Gf in 34 adult males. The authors showed that the statistical wavelet features and the wavelet coefficient features from the frequency bands 0.0–1.875 and 1.875–3.75 Hz resulted in 100 and 98% prediction accuracies, respectively (Qazi et al., 2017). However, the sample sizes of these studies were restricted to no more than 40, and the EEG evaluation index was limited to only one. Additionally, in the field of EEG markers of Gf, there are few comparable quantitative analysis studies, and the discriminant effect is easily affected by the discriminant method. Therefore, it is not robust enough to arrive at a conclusion; further exploration and verification are warranted. It is worth noting that while there was less discussion on the evaluation of EEG indicators in the studies of Gf, in other research fields such as mild cognitive impairment (MCI) and consciousness, the discrimination effect of EEG indicators has been discussed more fully. Missonnier et al. (2006) showed that the theta event-related synchronization (ERS) during the *n*-back working memory (WM) task can distinguish progressive MCI cases whose θ-ERS power was lower than that in the stable MCI cases, and the Area Under Curve (AUC) was 76% (Missonnier et al., 2006). While adding the event-related potential (ERP) index (P200 and N200), the combination model showed a higher AUC reaching 0.938 (Missonnier et al., 2007).

A relationship between WM and Gf has been well established. WM might be fractionated into two components: short-term memory (STM) storage and the central executive (CE). The CE is a processing component, which may be fractionated further into executive functions (EFs) like updating, inhibition, and shifting: the updating of information temporarily memorized for processing, the inhibition (or interference control) of information not or no longer relevant for the current processing step, and the shifting of the attentional focus between different task demands. Accordingly, WM-load can be differentiated into WM storage-load and WM processing-load (i.e., demanding STM processes and demanding EFs, respectively). A typical task to induce WM storage-load is the simple digit span (Dspan) task (i.e., the short-term memorization of a sequence of digits for later recall). In contrast, complex span tasks like *N*-back (*N* ≥ 2) tasks are conceptualized to induce WM processing-load (Engel De Abreu et al., 2010; Scharinger et al., 2017). Notably, even though STM and WM are theoretically distinct and sometimes assessed separately, no single task is a pure measure of either of them; even a seemingly simple task, such as Dspan, is likely to involve EFs mechanisms (Engel De Abreu et al., 2010).

The relationship between these two components of WM and Gf can be summed up using three kinds of views (mainly from the studies of structural equations and path analysis). First, STM system (the storage component of WM) has a particularly important connection with general intelligence (Colom et al., 2005; Gignac et al., 2016). Second, CE function plays a major role in Gf (Gray et al., 2017; Myers et al., 2017). Third, both STM (storage function) and WM (EF) are related to intelligence, and both components produce independent contributions to Gf, respectively (Unsworth and Engle, 2007; Unsworth et al., 2014). In all, none of them has completely denied the effect of WM processing or storage components on Gf; moreover, the relationship among them at the EEG level is still unclear. So, we assume that both the storage component (STM) and the non-storage component (EF) of WM affect Gf, and then we choose two typical representative tasks: 2-back for EF and delay match to sample (DSM) for STM in the present study.

Nevertheless, whether there exist EEG markers indicating Gf robustly is still dubious. Neural efficiency hypothesis and attentional resources allocation give cues that P3 amplitude induced by executive function task and θ-power in simple memory task may have the ability to indicate Gf. The neural efficiency hypothesis stated that brighter individuals display lower (more efficient) brain activation while performing simple cognitive tasks (Neubauer and Fink, 2009), and Julie et al. (2005) showed that the frontal midline θ-power increased as the memory load increased (Julie et al., 2005), suggesting that the frontal midline θ-power may indicate the amount of cognitive resources that need to be invested in current memory tasks, thus reflecting the subjective sense of task difficulty. Attentional resources allocation illustrated that P3 amplitude at parietal sites in the complex tasks would reflect the amount of attentional resources allocation that one person concentrates on current EF task (Polich, 2007). So, increased P3 amplitude is a manifestation of sufficient cognitive resources (Scharinger et al., 2017), and it would be accompanied by a better *N*-back performance (Tusch et al., 2016). In addition, neural speed is considered to be an evaluation index of cognitive ability, while P2 component is considered to reflect processes involved in selective attention (Wongupparaj et al., 2018) and shorter P2 latency is considered to reflect more shifting ability, which indicates more efficient use of brain resources (Lijffijt et al., 2009; Wongupparaj et al., 2018). So, we hypothesize that in the same simple memory task, children with high Gf would exhibit lower frontal midline θ-power (saving brain resources due to an easy feeling toward the task), and that in the EF task, they would exhibit larger parietal P3 amplitude (more attention resources can be focused on the task) and shorter P2 latency (more flexible) than that of children with low Gf. The present study intends to explore whether WM task-related EEG biomarkers can diagnose Gf level and predict academic achievement in healthy primary school children.

## Materials and Methods

### Subject

For the experiment, a sample of 62 healthy students (28 male; all right-handed; age range, 9–12 years) were recruited from a primary school in Nanning, China. They had normal or “corrected to normal” vision and were free from medication, neurological disorders, and cognitive impairments. Their parents all signed informed consent forms before the children participated in the trials. This study was approved by the Psychology Experimental Ethics Committee of Nanjing University.

### Raven’s Standard Progressive Matrix (RSPM) Test

The RSPM test was performed for intelligence assessment, during which 60 items were completed within 40 min. The original scores were calculated by adding up the scores for completing 60 items, which were converted into standardized intelligence scores (RSs) ranging from 0 to 100, and according to the Intelligence Level Grading Standard, intelligence levels were classified into the following five grades: 1 (>95, very good), 2 (95–75, good), 3 (74–25, average), 4 (24–5, below average), and 5 (<5, deficit) (Wang et al., 2009).

### WM Tasks

**FIGURE 1:**
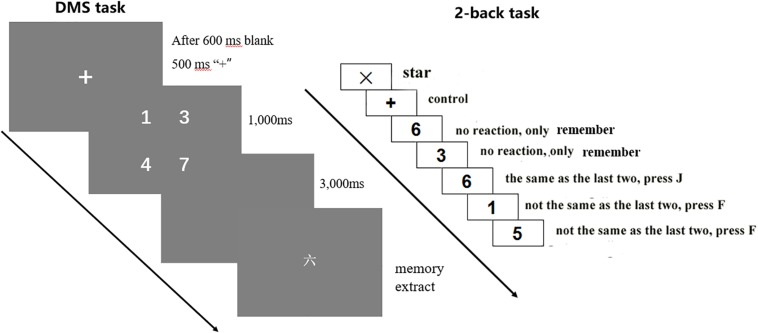
Working memory task.

DMS task is used to assess children’s ability related to encoding and storing information in STM. In general, the WM capacity of DMS paradigm is set at 4 (Zhang et al., 2016); so, the WM load in the present experiment was also 4. That is, four digits appeared each time. The task process is shown in Figure 1 (left): First, four Arabic numerals (1,000 ms) appear on the screen. The subjects are asked to remember the four numerals. Then, a blank screen of 3,000 ms appears. Finally, a capitalized numeral appears. The subjects are asked to react immediately to determine whether the current number was contained in the four numerals that just appeared. Contained, press “F” key, while not contained, press “J” key. The task includes two blocks; each block has 20 trials, making a total of 40 trials.

The 2-back task was applied to examine the children’s EF of WM. Apart from updating the WM content, when doing the task, the subjects must shift between the two subtasks and inhibit currently irrelevant information (Scharinger et al., 2017); so, it’s a complex task that requires all EF subcomponents (including inhibition, updating, and shifting). The instructions are shown in Figure 1. After reminding the subjects with “+,” an Arabic numeral in the range of 1–9 will appear randomly around the “+” for 600 ms. The subjects were required to compare whether the current number matched the number that was shown two numbers prior, by pressing the “J” key for matching, or by pressing the “F” key for mismatching. Each number after the third number should be judged one by one. Matching and mismatching conditions accounted for half of the trials. In order to ensure that the subjects understand the task, an exercise session was set before they entered the formal experiment. Only those with the correct rate of exercises reaching up to 60% can enter the formal test. The exercise is set to ensure that the participants understand the task; so, if they did not pass the exercise the first time, they can get a second and even a third opportunity to exercise again (no more than three exercise in all). In this research, all of the participants got through the exercise no more than three times.

There are two blocks, 40 trials in each block, and 80 trials in total. These two tasks were all programed in E-Prime 2.0, and each task was presented on a 16-inch computer screen from a height of horizontal line of sight.

### Experiment Procedure

All of the participants were informed of the schedule for data collection and, as per their availability, the experiments were arranged individually. Each subject was seated in a partially sound-attenuated room and was briefed on the procedure. Each subject was asked to perform the RSPM pencil-and-paper test first; next, each subject went to the nearby EEG room to perform the DMS task; and finally, they performed the 2-back task. The subjects had a 3-min break between tasks. During the WM time, an EEG cap was set until they completed the two computer tasks.

By the end of the next term (6 months later), the Chinese and mathematics scores of those subjects were collected as an index for academic achievement. The examinations test the students’ mastery of knowledge acquired in a semester, and the items are designed by teachers who teach the corresponding curriculum. The original scores of the examinations were transformed into *Z* scores according to the calculating formula: $z=\frac{\mathrm{original}\,\mathrm{score}-\mathrm{average}\,\mathrm{score}}{\mathrm{standard}\,\mathrm{deviation}}$, and the average score and the standard deviation values corresponded to the subjects’ grades to which they belonged.

### Electrophysiological Recordings

When the participants were performing the WM tasks, the EEG data were recorded using an EEG amplifier (NuAmps 40, Compumedics Neuroscan, VIC, Australia). The sample rate was set to 1,000 Hz with a bandpass filter (0.05–100 Hz), and the reference electrode was situated on the left mastoid online, and the grounding electrode was located at the midpoint of connection between FPz and Fz (called AFz). Horizontal eye movements were recorded by electrodes positioned at the outer canthus of each eye whereas vertical eye movements were recorded by electrodes positioned above and below the left eye. The electrode impedance was maintained at <10 kΩ throughout the EEG recordings. To attenuate low- and high-frequency noise, the averaged waveforms were filtered using a 30-Hz low-pass filter and a 0.5-Hz high-pass filter in the off-line analysis.

#### Preprocessing

Preprocessing was conducted using Curry 7.0 (Compumedics Neuroscan), including re-reference, removing EOG artifacts, deleting bad block. and segment epoch. This procedure is described as follows:

(1) Re-reference: change the reference from left mastoid to bilateral mastoid.
(2) Remove EOG artifacts: set the removing-threshold at 150 mV, removing EOG artifacts (which are above the threshold value) from the EEG signals based on a covariance method.
(3) Delete bad block: set the delete threshold at ±100 mV to exclude the impact of bad block in the next averaged waveforms step.
(4) Segment epoch: for 2-back data, the artifact-free EEG was segmented into epochs ranging from 200 ms before stimulus onset to 800 ms after stimulus onset, with a period of “–200 ms to 0” as baseline correction, fewer than 40 of 80 good target segments were excluded in the data analysis. For DMS data, the artifact-free EEG was segmented into epochs ranging from 500 ms before stimulus onset to 4,000 ms after stimulus onset, according to the task design; the 0–1,000 ms was coding period, 1,000–4,000 ms was delay period at each epoch, and “–500 ms to 0” was used as baseline correction. Fewer than 20 of 40 good target segments were excluded in the subsequent data analysis.

### Data Analysis

#### Behavioral Analysis

Behavioral data were analyzed to measure performances corresponding to fluid cognitive ability as well as the WM tasks. To assess fluid cognitive ability, RSPM raw scores and standardized intelligence scores, as well as the intelligence level, were calculated for each subject. Considering that there were no children with deficits in our study sample and that the “very good” and “below average” levels were also not sufficient to form an independent group, we combined the “very good” and “good” children into the HA group and the “average” and “below average” children into the LA group. It should be noted that in similar studies by Amin et al. (2015) and Qazi et al. (2017), in which they grouped adult subjects according to the median scores of RAPM raw scores, those who scored above the median were placed in the HA group, and conversely, those who scored below the median were placed in the LA group. But in children, age is a notable factor that would affect the Raven raw scores; so, if we do like this in the present experiment, most of the older children might be grouped in the HA group. Therefore, in order to prevent this issue, we use the intelligence level that has already considered Raven’s score and children’s age at the same time.

For the WM tasks, each subject’s performance was computed by calculating the number of correct responses (accuracy, ACC) in addition to reaction time (RT). Independent sample *t*-test was used to analyze the data with ACC and RT. A statistical analysis was performed using SPSS version 22.0 software (IBM, China).

#### ERP and ERS Analyses

For 2-back EEG data, a superimposed averaging process can be carried out after preprocessing; only good segments were retained in the individual averaged waveforms. In addition, to investigate whether the differences between the two groups are specific to P3 only, the P2 component was extracted and analyzed. For DMS EEG data, before obtaining the superposed average, a wavelet transform was applied to extract theta power. Both the wavelet transform and the superposed average were conducted using MATLAB R2013b, with toolbox Letswave7^1^.

For ERP analysis, the waveforms and the 2-D plot of group grand average were performed before determining the time window of ERP components; the major electrodes were selected in the groups (HA vs. LA) × electrode sites (*n*) repeated measurements analysis of variance (ANOVA) test; both ERP amplitude and latency were extracted from the respective electrodes for each subject per group. For ERS analysis, the time-frequency map and the 3D-plot of group grand average were conducted before selecting the representative electrode sites. Also, the θ-power of the selected electrodes were analyzed by repeated measurements of variance of 2 (grouping: HA vs. LA)× *n* (electrode sites). Greenhouse–Geisser method was used to correct the *p* value while the statistical results were not satisfied with the spherical assumption, and Bonferroni method was used to correct multiple comparisons (*n* times) afterward. The ANOVA test was conducted using SPSS 22.0 software.

#### Logistic Regression and SVM

Logistic regression (LR) and SVM were two major classifiers that were applicable for non-linear discriminant analysis. The LR was based on probability theory [see Function (1), the samples that indicate *P* > 0.5 would be considered to be positive ones; a positive event here refers to LA], whereas the SVM is based on maximizing geometric interval [see Function (2)–(5)]; thus, the optimal hyperplane found by the LR model is to try to keep all of the sample points away from it, and the optimal hyperplane that the SVM is looking for is to maximize the margins (keep only the training points closest to the boundary line as far as possible). So, in the LR model, each sample data would affect the result, whereas in the SVM model, only the samples near the boundary line (that is, only those samples that support the vector) would be considered. Because of the data limitations, the *kernel* SVM was chosen as classifier instead of linear SVM. It projects implicitly the feature of low dimensionality to high dimensionality, and makes the feature disentangled in high dimensionality.

$$P\left(Y=1|x\right)=\pi_{x}=\frac{e^{\beta_{0}+\beta_{1}\,x}}{1+e^{\beta_{0}+\beta_{1}\,x}}$$

*Y*: intelligence group (1: LA, 0: HA); *x*: EEG markers; β_0_: the constant; β_1_: The estimated coefficient of *x*; *P*(*Y* = 1|*x*): Given the *x*, the probability that an individual belongs to the LA group.

As described above, the hyperplane in *kernel* SVM can be described as follows:

$$f\,\left(x\right)=w^{T}\,\varphi \,\left(x\right)+b$$

And the radial basis function is:

$$k\,\left(x_{i},x_{j}\right)=\exp\left(-\frac{{\left|x_{i}-x_{j}\right|}^{2}}{2\,\sigma^{2}}\right)$$

where σ is the width of kernel function; usually, $\frac{1}{2\,\sigma^{2}}$is called gamma factor. It assumes that all of the samples are separated, and subjects to the inequation as follows:

$$y_{i}\,\left(w^{T}\,x_{i}+b\right)\gg 1$$

In practice, not all of the samples can be separated precisely by hyperplane. In order to reduce the influence of these special undesired samples, the approach of soft margin is introduced to SVM. It allows the samples to classify the opposite category in some degree:

$$y_{i}\,\left(w^{T}\,x_{i}+b\right)\gg 1-\xi_{i}$$

where *ξ*_*i*_is slack variable, representing the degree of every sample that deviates from the accurate category. In the phase of optimization, C will be introduced to control the degree of fitting.

In the present study, we used the “tune” parameter sweep tool [R coding: tune (SVM, Group∼, data = IQ_train, kernel = “radial,” ranges = list (cost = c(0.001, 0.01, 0.1, 1, 10, 100, 1000)))]. A grid search was performed on seven parameter values between *C* = [10^–3^ to 10^3^] on the whole data. This suggested values of *C* = 1 (which let the model reach its least error: 0.27). In addition, the gamma is set to 1/*N*_*f*_ (*N*_*f*_ represents the feature of dimensionality). Those parameter values were used for the subsequent analysis.

In order to obtain more compelling results, We adapted fourfold cross-validation, which separated data into four segments: three for training and one for testing [75% for training and 25% for testing, leaving sufficient testing sample to ensure that it can provide useful information about accuracy rate (Stewart et al., 2014)]. Iterating through the cross-validation, each subset was used once as test data, and the score was averaged across the four splits. Additionally, to ensure comparability between these two models, an R code “set.seed(20)” was written before the cross-validation part to ensure that the division of sets was exactly the same between each model. Relatedly, the *caret*^2^, *glm* and *e1071*^3^ packages of the R Studio software version 1.1.456 were utilized to conduct the corresponding tests (i.e., *glm* for logistic model testing; *e1071* for SVM testing; and *caret* for cross-validation).

Besides, the receiver operating characteristic (ROC) technique was adopted for evaluating the LR and SVM models [for more details about the ROC technique, see Fawcett, 2006; Hand, 2009]. An ROC plot illustrates both sensitivity and specificity with the AUC of the ROC of 0.5 signifying random chance prediction and 1 being perfect prediction. Therefore, the closer the AUC is to 1, the greater the diagnostic value of the indicator(s). The *pROC* packages^4^ of the R Studio software version 1.1.456 were utilized to plot the ROC curve.

#### Multiple Linear Regression (MLR) Model

The MLR is a linear statistical method, which is used for predicting the relationship of a single dependent variable (response variable: *Y*) with one or more independent variables (predictors: *X*1, *X*2, …, *X*n). A general MLR model can be described by the following equation:

$$y=\beta_{0}+\beta_{1}\,x_{1}+\beta_{2}\,x_{2}+\cdots +\beta_{n}\,x_{n}+\varepsilon$$

where *Y* represents the dependent variable, *x*_*i*_indicates the *i*_th_ independent variable, β_*i*_represents *i*_th_ predicted parameter (regression weight), and ε is the error between predicted response and observation. The regression weights (β_*i*_) are computed in such a way that minimizes the sum of squared deviations.

In this study, the MLR analysis was performed using SPSS 22.0 with “enter” method on the selected EEG index with selected electrodes to predict academic achievement (*Y*). Before performing the regression, we had to decide which variable should be used in the regression model. The method included “enter,” “remove,” “forward,” “backward,” and “stepwise.” We selected “enter” method to let all the Xs enter the model to test their determinant coefficients. To evaluate statistically the LR model, the following important assumptions about the residuals were considered and verified (Amin et al., 2015):

(1) The residuals should have zero mean value (Linearity).
(2) The residuals should be plotted as normal distribution (Normality).
(3) The residuals should have constant variance (Homoscedasticity).
(4) The residuals are independent (or random); otherwise, autocorrelation problem exists.
(5) The Xs are independent; otherwise, multicollinearity problem exists.

Assumption (1) is easily verified by Residual Frequency Distribution Map (see Figure 9C in the Results). And if a normal probability plot of the standardized residuals showed a straight line, assumption (2) is verified. Assumptions (3) and (4) can be evaluated by using scatter plots that show the relationship between standardized residuals and predicted values. Besides, the variance inflation factor (VIF) is introduced to detect the LR model collinearity with a threshold at 10 to verify assumption (5). The verification of these assumptions is given in the section “Verification of Regression Assumptions.”

## Results

According to the participants’ RSPM scores, eight subjects were rated as “very good,” 34 as “good,” 17 as “average,” and 3 as “below average”; so, 42 children were assigned to the HA group and the rest were assigned to the LA group. Sex distribution has shown non-significant difference between the two groups (*x*^2^ = 1.154, *p* = 0.413). The grouping information is presented in Table 1.

**TABLE 1:** Grouping results (mean ± standard deviation).

	**HA**	**LA**	***t***	***p***
	**(*N* = 42, 17 male)**	**(*N* = 20, 11 male)**		
Age	10.94 ± 0.66	11.18 ± 1.07	–1.067	0.290
Raw scores of RSPM	48.29 ± 3.73	37.55 ± 5.51	9.036	<0.001

### Behavioral Results

Behavioral data recorded during the DSM and the 2-back task were analyzed for both groups (HA and LA). As shown in Table 2, the HA group’s accuracy (ACC) was significantly (or marginally significant) higher than the LA group’s ACC for both tasks, while the HA group’s reaction time (RT) was shorter (non-significantly) than the LA group’s RT. Additionally, Cohen’s *d* results (Table 2) indicated an intermediate effect size between the HA and the LA groups’ performances for the ACC.

**TABLE 2:** Working memory performance measurements (mean ± standard deviation).

	**HA (*N* = 42)**	**LA (*N* = 20)**	***t***	***p***	**Effect size (Cohen’s *d*)**
ACC of DMS	0.90 ± 0.07	0.84 ± 0.11	2.632**	0.011	0.651
RT of DMS (ms)	832.46 ± 96.56	807.14 ± 110.57	0.921	0.361	0.244
ACC of 2-back	0.84 ± 0.10	0.78 ± 0.12	1.989*	0.051	0.543
RT of 2-back (ms)	1025.25 ± 327.33	935.72 ± 292.47	1.041	0.302	0.288

*p < 0.10 mark; **p < 0.05. Small effect, 0.15 ≤ *d* < 0.40; medium effect, 0.40 ≤ *d* < 0.75; large effect, 0.75 ≤ *d* < 1.10; very large effect, 1.10 ≤ *d* < 1.45; huge effect, *d* > 1.45.

### ERP Results

**FIGURE 2:**
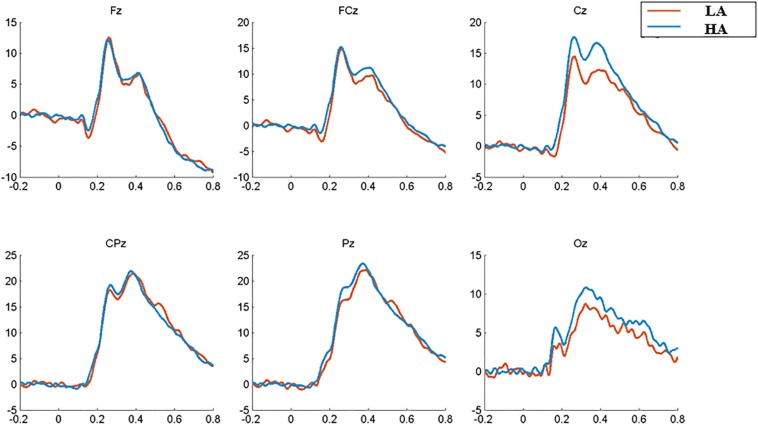
Average ERP waveforms for 2-back task of LA (red) and HA (blue) groups.

**FIGURE 3:**
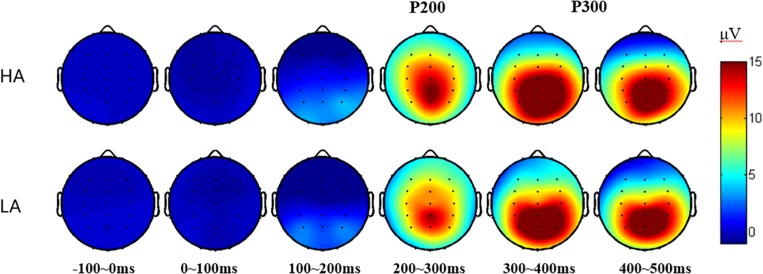
2-D plot of grand average ERP responses of HA and LA groups from 34 scalp locations (except for four eye electrodes and two reference sites).

The subjects were excluded from further ERP analysis due to an insufficient number of target segments (fewer than 40 of 80 good target segments) that failed to obtain adequate “signal to noise ratio.” This exclusion allowed 60 subjects for 2-back ERP analysis and excluded two subjects. With regard to waveform and 2-D topographic map (Figures 2, 3), the time window of P2 is set at 220–280 ms, and P3 is set at 350–420 ms.

The analysis of latency showed that the Fz site reaches the P2-peak first (around *t* = 0.26 s) and that the Pz site reaches the P3-peak first (around *t* = 0.37 s); so, the comparison of the latency between LA and HA group is conducted for P2(Fz) and P3(Pz), respectively. The results revealed a marginally shorter (*t* = 1.783, *p* = 0.080, Cohen’s *d* = 0.497) P2 (Fz) latency of the HA group (253.55 ± 12.75 ms) compared to that of the LA group (260.06 ± 13.43 ms). For P3(Pz) latency, a non-significant difference has been found [HA: 371.9 ± 33.0 ms; LA: 386.1 ± 38.7 ms, *t* = 1.492, *p* = 0.141, Cohen’s *d* = 0.395].

Following previous research (Amin et al., 2015; Zhang et al., 2018) and based on our total average results (Figure 3), the electrode sites that show P200 or P300 component are used in further analysis (see Tables 3, 4, respectively); so, a 2 (group: HA and LA) × 5 (sites: Fz, FCz, Cz, CPz, and Pz) repeated measures ANOVA was performed to analyze the average amplitude of the P200, and a 2 (group: HA and LA) × 5 (sites: FCz, Cz, CPz, Pz, and Oz) repeated measures ANOVA was performed to analyze the average amplitude of the P300.

**TABLE 3:** P200 amplitude of each group in 2-back task (μV, mean ± standard deviation).

	**HA (*N* = 42)**	**LA (*N* = 18)**
Fz	11.58 ± 5.27	12.43 ± 5.09
FCz	14.72 ± 5.67	14.81 ± 4.64
Cz	17.09 ± 5.79	14.81 ± 7.05
CPz	18.36 ± 6.22	17.89 ± 4.72
Pz	17.26 ± 7.51	15.93 ± 6.48

**TABLE 4:** P300 amplitude of each group in 2-back task (μV, mean ± standard deviation).

	**HA (*N* = 42)**	**HA (*N* = 18)**
FCz	11.32 ± 6.04	9.85 ± 7.19
Cz	16.77 ± 6.20	12.87 ± 8.30
CPz	21.39 ± 6.40	18.93 ± 6.47
Pz	22.68 ± 7.36	21.04 ± 5.98
Oz	9.44 ± 7.30	7.53 ± 8.60

The results of repeated measures ANOVA of P2 amplitudes between these two groups indicated a significant main effect of electrode sites [*F*(4,232) = 19.948, *p* < 0.001, η^2^ = 0.256], Further multiple comparisons showed that the P2 amplitude in the central-parietal region (CPz) was significantly higher than that in the frontal region (Fz, *p* < 0.001), frontal-central region (FCz, *p* < 0.001), central region (Cz, *p* = 0.005), and parietal region (Pz, *p* = 0.023); and the P2 amplitude at Fz site was significantly smaller than that at other sites (*p* ≤ 0.001). The group’s main effect [*F*(1,58) = 0.196, *p* = 0.660, η^2^ = 0.003] and the interaction effect between groups and electrode sites [*F*(4,232) = 1.412, *p* = 0.231, η^2^ = 0.024] were not significant.

For P3 amplitude, the statistical results showed a significant main effect of electrode sites [*F*(4,232) = 55.074, *p* < 0.001, η^2^ = 0.487], revealing that the P3 amplitude decreased from Pz and CPz sites to Cz, FCz, and Oz sites, respectively; and a marginally significant group main effect was found [*F*(1,58) = 2.876, *p* = 0.095, η^2^ = 0.047]. Further multiple comparison indicated that the P3 amplitude in the HA group was significantly higher than that in the LA group at Cz site (*p* = 0.049). The interaction effect between electrodes sites and groups was non-significant [*F*(4,232) = 21.496, *p* = 0.702, η^2^ = 0.007].

### θ-ERS Results

**FIGURE 4:**
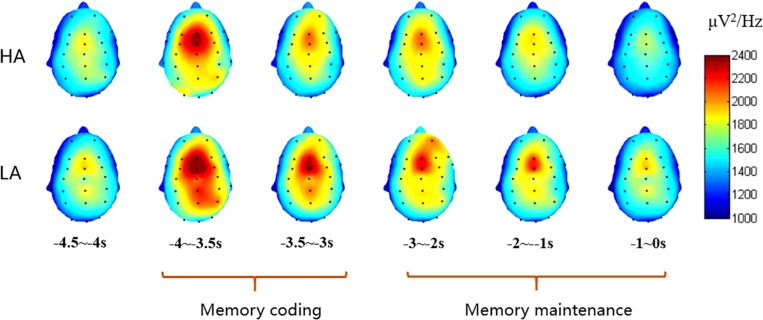
3-D plot of theta power for coding and delay periods of each group.

The subjects were excluded from further ERS analysis due to an insufficient number of target segments (fewer than 20 of 40 good target segments) that failed to obtain adequate “signal to noise ratio.” This exclusion allowed 58 subjects for DMS ERS analysis and excluded four subjects. With regard to the 3-D spectrogram (Figure 4), the major sites with obvious activity in the theta band were included in the repeated measures ANOVA.

Consistent with previous empirical studies like, Tóth et al. (2014) and Raghavachari et al. (2001), the theta band (4–7 Hz) activities were major in the frontal area. Then, a 2 (group: HA and LA) × 6 (sites: F3, Fz, F4, FC3, FCz, and FC4) repeated measures ANOVA was conducted to analyze the theta power between these two groups for coding and maintaining periods, respectively. The statistical results for coding period showed a significant main effect of electrode sites [*F*(5,280) = 31.693, *p* < 0.001, η^2^ = 0.361] and a significant interaction effect between electrodes sites and groups [*F*(5,280) = 2.649, *p* = 0.023, η^2^ = 0.045], while the main effect of group was non-significant [*F*(1,56) = 0.407, *p* = 0.526, η^2^ = 0.007]. A further simple effect analysis shows that the theta power of the LA group was marginally higher than that of the HA group at FCz site (*p* = 0.081). The statistical results of delay (maintaining) period indicated a significant main effect of electrode sites [*F*(5,280) = 21.251, *p* < 0.001, η^2^ = 0.275], while non-significant effects of group [*F*(1,56) = 0.522, *p* = 0.473, η^2^ = 0.009] and the interaction of group and electrode sites [*F*(5,280) = 1.785, *p* = 0.116, η^2^ = 0.045] were found. The theta power of these sites for each period of two groups are presented in Table 5.

**TABLE 5:** θ-ERS of each group in DSM task (Power, μV^2^/Hz).

	**HA (*N* = 39)**		**LA (*N* = 19)**	
	**Coding period**	**Maintaining period**	**Coding period**	**Maintaining period**
F3	1885.74 ± 654.33	1656.17 ± 611.50	1901.95 ± 704.60	1697.08 ± 618.10
FZ	2216.02 ± 653.01	1876.24 ± 616.25	2386.88 ± 567.21	2086.95 ± 531.09
F4	1841.89 ± 582.67	1600.36 ± 565.92	1804.06 ± 502.64	1639.04 ± 507.29
FC3	1857.27 ± 600.07	1625.65 ± 572.07	1836.67 ± 751.88	1617.21 ± 647.29
FCZ	2196.45 ± 645.42	1876.60 ± 605.67	2518.10 ± 649.40	2123.21 ± 519.66
FC4	1850.08 ± 575.04	1639.55 ± 565.17	1988.73 ± 414.47	1735.13 ± 343.39

**FIGURE 5:**
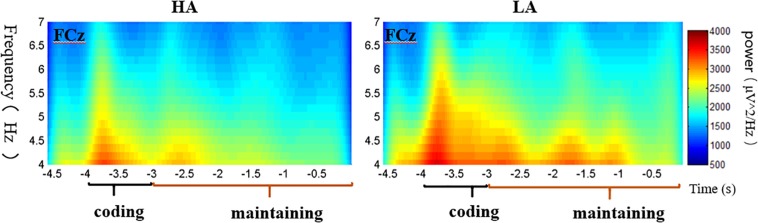
Average spectrograms at FCz site of HA **(left)** and LA **(right)** groups.

Taking FCz site as an example (Figure 5), the theta power increased at the commencement of the trial and was elevated through the memory coding period and the delay period. The average spectrograms for the LA group demonstrated more energy in the theta frequency band across the coding period.

### Machine Learning Results

**FIGURE 6:**
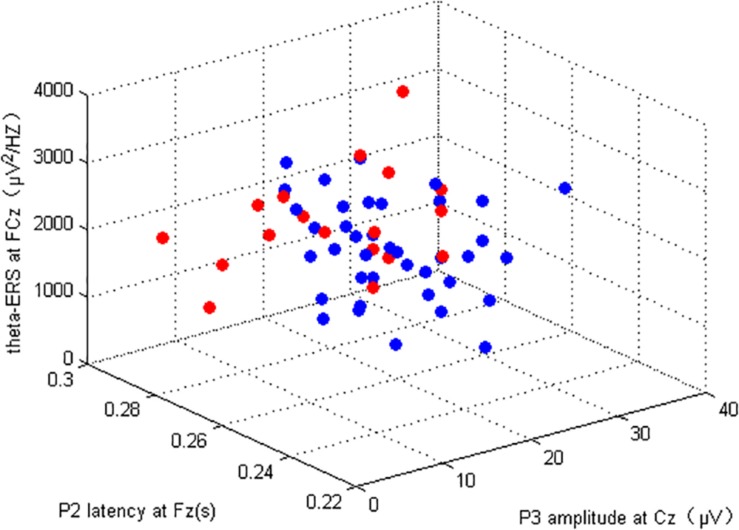
High ability (blue) and LA (red) individual location determined by three EEG indicators.

P3 amplitude (Cz), P2 latency (Fz) of 2-back task, and the θ-ERS (FCz) within the coding period of the DSM task were included in the machine learning analysis; the total number of subjects in this section was 56 (consider both 2-back and DSM tasks). The Gf (marked by RSPM) of our subjects was linearly inseparable by three EEG indicators (Figure 6). As illustrated in the section “Materials and Methods,” the classification performance of fourfold cross-validation of *kernel* SVM and LR classifiers for each EEG indicator and the combination of the indicators are presented in Table 6. The mean was the average of the testing results of fourfold cross-validation, as well as standard deviation values. Use of a single EEG parameter permitted correct classification of 76.8% (for P3 amplitude), 69.6% (for P2 latency and theta ERS) using the SVM model, as well as a combination of these three EEG markers of 73.2%, which was lower than the P3 amplitude. For the LR model, the correct classification of P3 amplitude is 69.6%, and the classifications of both P2 latency and theta ERS are 67.9%, a combination of them is 71.4%.

**TABLE 6:** Parameters of classification (mean ± standard deviation) of testing set.

	**SVM**			
	**P3 amplitude**	**P2 latency**	**Theta ERS**	**All**
Accuracy	0.768 ± 0.107	0.696 ± 0.122	0.696 ± 0.122	0.732 ± 0.107
Sensitivity	0.217 ± 0.208	0.000 ± 0.000	0.000 ± 0.000	0.104 ± 0.125
Specificity	1.000 ± 0.000	1.000 ± 0.000	1.000 ± 0.000	1.000 ± 0.000

	**LR**			
	**P3 amplitude**	**P2 latency**	**Theta ERS**	**All**

Accuracy	0.696 ± 0.122	0.679 ± 0.092	0.679 ± 0.092	0.714 ± 0.143
Sensitivity	0.167 ± 0.236	0.000 ± 0.000	0.000 ± 0.000	0.317 ± 0.281
Specificity	0.927 ± 0.086	1.000 ± 0.000	1.000 ± 0.000	0.864 ± 0.077

The accuracy of SVM was higher than that of LR classifier for both the single or combined EEG indicators, which again verified the good generalization capabilities of SVM algorithms based on maximizing the margin that Lotte et al. (2007) had previously mentioned. But it should be noted that with respect to the AUC of ROC, the LR model showed better outcomes, especially in the regression of three comprehensive indicators, reflecting its advantages of “taking care of the overall samples” which leads to an AUC at 0.844, which is higher than that of SVM (0.792), and far higher than any single EEG indicator in the LR model (AUC all < 0.6, almost equal to 0.5, which signifies random chance prediction). Among the three single EEG indicators, P3 amplitude was by far the more suitable indicator in the discrimination of Gf because of its highest accuracy rating in both classifiers. It is worth mentioning that while the specificity was excellent in the SVM classifier (single or comprehensive indicators have reached 1 for all four subsets), the sensitivity of diagnosing individuals with LA in both classifiers was very small, even < 0.5, indicating the limitation of those EEG signals.

**FIGURE 7:**
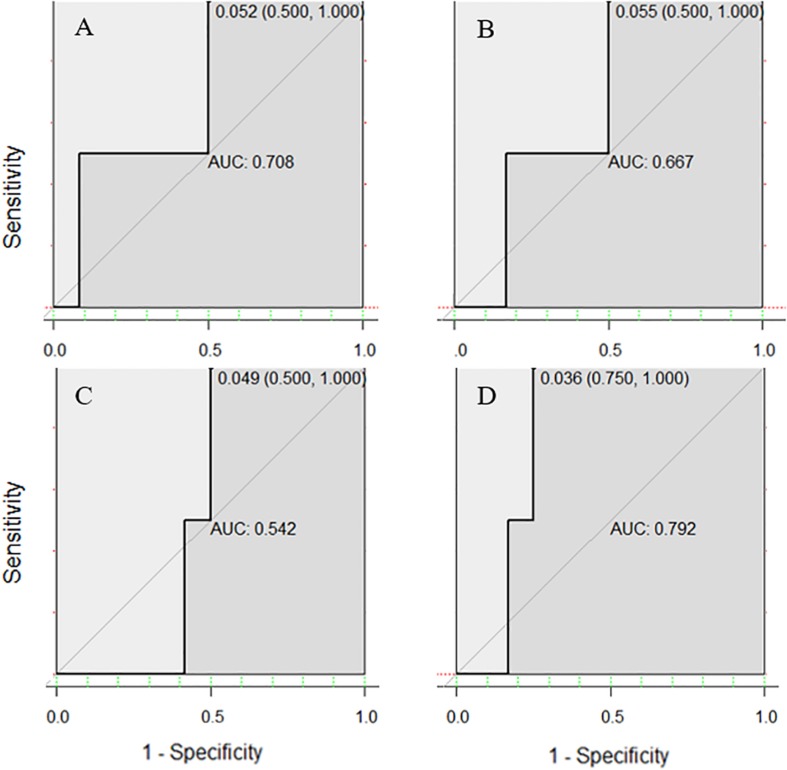
Receiver operating characteristic curves for two-back-related frontal P3 amplitude **(A)**, P2 latency **(B)**, and DMS-related theta ERS **(C)**, and their combination based on electrode sites selected from the ANOVA test **(D)** using the SVM model.

**FIGURE 8:**
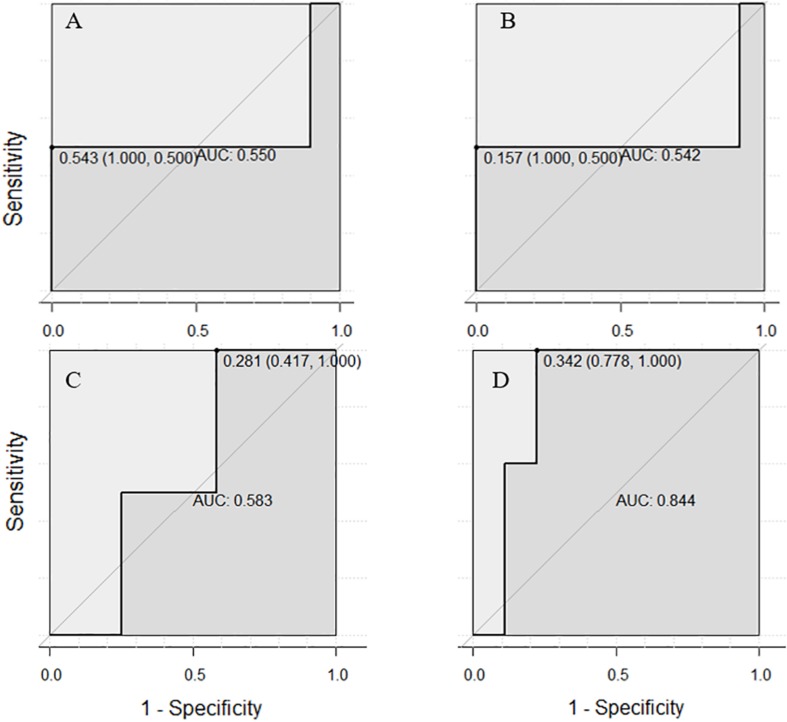
Receiver operating characteristic curves for two-back-related frontal P3 amplitude **(A)**, P2 latency **(B)**, and DSM-related theta ERS **(C)**, and their combination based on electrode sites selected from the ANOVA test **(D)** using the LR model.

The training sets that corresponded to the testing sets with the highest accuracy in both SVM and LR models were used to draw ROC curves. Interestingly, the area under the ROC curve of SVM and LR showed different styles. In the ROC curve under the SVM model (Figure 7), all of the best cutoff points were set at the point where sensitivity was equal to 1, and the shape of single or combined signals was similar to each other (all showed high sensitivity and low specificity). But in the ROC curve of the LR model, the combination of the three EEG indicators led to a substantial improvement of sensitivity (reach at 1.00), specificity (reach at 0.75), and proportion of correctly classified cases (Figure 8). Meanwhile, when the best cutoff points of P3 amplitude and P2 latency were set at the point where they had an advantage in specificity (equal to 1), the best cutoff point of θ-ERS had an advantage in sensitivity; so, it is reasonable to infer that their combination will demonstrate substantial improvement in AUC (under the LR model).

### Multiple Linear Regression Results

Multiple linear regression analysis with “enter” method was performed on selected three electrodes for P3 amplitude, P2 latency, and theta ERS for predicting academic achievement (6 months later). The regression parameter was presented in Table 7, and the regression function was described as follows. The P3 amplitude at Cz site predicted statistically and significantly the academic achievement (total scores of Chinese and Mathematics) in this model. The explanation ratio of variance between regression and residuals was marginally significant (*F* = 2.655, *p* = 0.058).

**TABLE 7:** Regression parameter of EEG signals to academic achievement.

**Independent variables**	***B***	**Standardized β**	***p***	**VIF**
θ ERS in coding period (FCz)	–0.000099	–0.150	0.262	1.002
P3 amplitude (Cz)	0.017**	0.285**	0.035	1.044
P2 latency (Fz)	–3.450	–0.113	0.384	1.046
Constant	1.716	7.382	0.220	–

*p < 0.10; **p < 0.05. VIF < 10 means that this variable does not have independent variable multicollinearity. The regression function: Academic Score = 1.414 − 0.000099 θ power + 0.017 P3 Amplitude − 3.540 P2 latency.

#### Verification of Regression Assumptions

**FIGURE 9:**
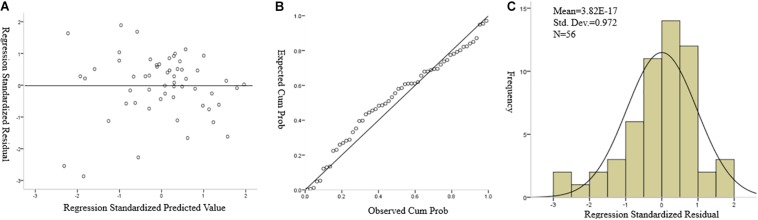
**(A)** Scatter plot of regression standardized residual against the regression standardized predicted value of dependent variable (academic score). **(B)** Normal P–P plot of regression standardized residual, the plot of the residual fits the expected pattern well enough to support that the residual is normally distributed. **(C)** Normal distribution plot of regression standardized residual with zero mean value.

With regard to the regression analysis for prediction of academic scores, the mean value of the residual is about 3.82 × 10^–17^, which is very close to zero (Figure 9C), and it also presents a normal distribution for the standardized residual; thus, the first and second regression assumption (linearity and normality) is verified. Besides, the normal probability plot of the standardized residuals shows a straight line that verifies the second assumption again (Figure 9B). The VIF of each independent variable is lower than 10, which shows that there is no multicollinearity problem in the regression model. The scatter plot of the residual against the predicted variable (Figure 9A) shows no specific pattern that can be observed, hence verifying the third assumption (constant variance, or homoscedasticity) and the fourth assumption (independence). Thus, the regression model assumptions are considered verified.

## Discussion

This study details a method for classifying Gf level in children using WM task-related EEG signals relying on machine learning. It also investigates the relationship between individual differences in WM task-related EEG signals and academic achievement. The present data suggest that P3, which reflects attentional processes involved in stimulus processing and inhibitory control, may be a biomarker for academic achievement during childhood, supporting in part what Hillman et al. (2012) had previously mentioned. To the best of our knowledge, this study is the first to apply pure EEG variables as independent variables to predict academic scores in a multiple linear regression model with verification, although the overall explanatory power is not strong (the explanation ratio of variance between regression and residuals was only 2.655); this research supplements the current literature: Although several studies demonstrated a significant connection between EEG signals measures and Gf, e.g., spectral power (Qazi et al., 2017) or P3 amplitude (Amin et al., 2015), in which the AUCs were >0.80, the present data in children could not support such connections.

The present results offer three implications: The first implication concerns experimental object. The discriminant analysis conducted in healthy people often does not demonstrate significant differentiation; not only in the results of the ROC but also in the analytical results of repeated measures ANOVA or *T*-test can we see that the differences between the HA and the LA groups of the three EEG indicators are only marginally significant. This may help to explain why some studies like the one by Covey et al. (2019), whose aim was to improve Gf in healthy groups, showed little change in the Raven’s scores, while the change in EEG signals yielded a significant training effect, as reported previously in a meta-analysis by Melby-Lervåg et al. (2016). The EEG signals did not appear to be so sensitive in the assessment of Gf, especially when Gf was evaluated using the pencil-and-paper test and Raven’s scores. Thus, we can infer from the present study that there may have been two possible reasons related to this phenomenon. One is that the EEG signals actually have little in common with the pencil-and-paper test; that is, the EEG signals change a lot, whereas the pencil-and-paper scores do not, or vice versa. If so, there will again be the challenge to determine what Gf is. Do the current tests based on the pencil-and-paper test really measure Gf? The other one is that in healthy samples, the difference between EEG signals and their Gf was too small to reach an acceptable sensitivity, at least based on the present method. So, if those methods were applied to the clinical samples with intellectual impairment in which the difference between positive and negative patients is large enough, the effect size would be greater.

Second, from the comparison of SVM and LR in the present study, we can summarize that while the accuracy of SVM was higher than the LR, the AUC of ROC in the LR model showed a larger AUC for the combined EEG signals, under the condition that the training and testing sets were the same between these two models. Additionally, it should be noted that although several studies have found that the indicator effect of the comprehensive indicator was better than the single indicator (Missonnier et al., 2007; Engemann et al., 2018), a counterexample appeared in this study, indicating that using one signal of P3 amplitude as input increases the classification accuracy to 76.8 from 73.2% with three complexes in the SVM model. There is speculation that the *kernel* SVM projects implicitly the feature of low dimensionality to high dimensionality and makes the feature disentangle in high dimensionality; so, adding features may cause redundancy rather than improve accuracy. In addition, every single indicator in the LR model showed only a small AUC just above chance (the AUC is close to 0.5), indicating that the LR method is sensitive to outliers; meanwhile, a complementary effect has been found between ERP and ERS in the LR model, verifying that the selection of indicators is comprehensive and that it contributed to the improvement of the AUC in the combined indicators.

Finally, starting from the relationship between WM and Gf, this study essentially analyzes the WM task-related EEG signal and other EEG signals like resting state EEG signal (Gordon et al., 2018), functional connection signals, and other task-related EEG indicators that warrant further investigation in future studies. If there are EEG markers that can robustly indicate human Gf – no matter in what forms – current test styles for assessing intelligence could change dramatically.

Altogether, these findings extend and challenge previous findings that reported EEG signals might be used as a supporting factor in standard psychometric tests to assess an individual’s IQ. We hope that the present work, as well as recent studies, will motivate researchers to further explore these important concerns.

### Limitations

As we tried to control the experiment object and operation process, there are still some aspects that can be improved. First, we included children of different ages because we could not recruit a sample of children of the same age; when we grouped the children, we did consider this limitation. However, if we use a sample of children of the same age, or perhaps different age groups, the study would undoubtedly be stronger. Second, this study only focuses on three WM-related EEG candidate indicators. Although the WM is thought to be a complex system, perhaps there will be a more comprehensive EEG index system to reflect the WM in the future. Third, when we considered the predictive ability of EEG index, we only perceived it within the “WM-related” scope in the present study, but other EEG signals showed a significant correlation to academic performance, such as error-related negativity (Hirsh and Inzlicht, 2010). Therefore, future research might explore a more intense or broader scope.

## Data Availability Statement

The datasets generated for this study are available on request to the corresponding author.

## Ethics Statement

The studies involving human participants were reviewed and approved by the Psychology Experimental Ethics Committee of Nanjing University. Written informed consent to participate in this study was provided by the participants’ legal guardian/next of kin.

## Author Contributions

WL and RZ came up with the idea. RZ and WL designized the experiment and modified the final manuscript. WL collected the data and prepared the draft.

## Conflict of Interest

The authors declare that the research was conducted in the absence of any commercial or financial relationships that could be construed as a potential conflict of interest.

## Footnotes

1 For more details, see https://letswave.cn/index.html

2 Available at https://cran.r-project.org/web/packages/caret/index.html

3 For more details, see https://cran.r-project.org/web/packages/e1071/vignettes/svmdoc.pdf

4 Available at https://cran.r-project.org/web/packages/pROC
